# Influence of Chlorhexidine Gluconate on the Immediate Bond Strength of a Universal Adhesive System on Dentine Subjected to Different Bonding Protocols: An In Vitro Pilot Study

**DOI:** 10.3290/j.ohpd.a43934

**Published:** 2020-04-01

**Authors:** Gabriela Lopes Fernandes, Henrico Badaoui Strazzi-Sahyon, Thaís Yumi Umeda Suzuki, André Luiz Fraga Briso, Paulo Henrique dos Santos

**Affiliations:** a PhD student, Department of Dental Materials and Prosthodontics, Araçatuba School of Dentistry, São Paulo State University – UNESP, Araçatuba, SP, Brazil. Performed the experiments for the study; wrote the manuscript.; b PhD student, Department of Dental Materials and Prosthodontics, Araçatuba School of Dentistry, São Paulo State University – UNESP, Araçatuba, SP, Brazil. Performed the experiments for the study; wrote and proofread the manuscript.; c Professor, Department of Restorative Dentistry, Faculty of Dentistry, Federal University of Minas Gerais - UFMG, Belo Horizonte, MG, Brazil. Contributed to the idea, hypothesis and experimental design of the study.; d Associate Professor, Department of Restorative Dentistry, Araçatuba School of Dentistry, São Paulo State University – UNESP, Araçatuba, SP, Brazil. Contributed to the idea, hypothesis and experimental design of the study.; e Associate Professor, Department of Dental Materials and Prosthodontics, Araçatuba School of Dentistry, São Paulo State University – UNESP, Araçatuba, SP, Brazil. Contributed to the idea, hypothesis and experimental design of the study; performed the statistical analysis; wrote and proofread the manuscript.

**Keywords:** adhesives, dentine, microscopy electron scanning, resin composites, tensile strength

## Abstract

**Purpose:**

The aim of this in vitro study was to evaluate the influence of preapplication of 2% chlorhexidine gluconate on the immediate microtensile bond strength of a universal adhesive system on dentine subjected to different bonding protocols.

**Materials and Methods:**

Twenty human molars were used in this study, and the tooth surface was abraded to expose the dentine. The teeth were randomly divided into four groups according to the surface treatment (n = 5): SBU group: Single Bond Universal without acid etching; SBUPA group: 37% phosphoric acid + Single Bond Universal; SBUCG group: 2% chlorhexidine gluconate + Single Bond Universal; and SBUPACG group: 37% phosphoric acid + 2% chlorhexidine gluconate + Single Bond Universal. The microtensile bond strengths were measured using a microtensile tester 24 h after bonding. The bond strength data were subjected to analysis of variance (ANOVA) and Sheffé’s least statistically significant difference test (α = 0.05).

**Results:**

No statistically significant differences between the analysed groups were observed (p > 0.05). However, conditioning with phosphoric acid without the action of the chlorhexidine gluconate group resulted in higher numerical values of bond strengths than that for the chlorhexidine gluconate without the acid conditioning group.

**Conclusion:**

The preapplication of 2% chlorhexidine gluconate did not reduce the immediate bond strength of the Single Bond Universal adhesive system under different bonding protocols.

The bond stability of the adhesives on dentine substrates is fundamental to ensure the success of oral rehabilitation. Despite the significant advances made in this field, the longevity of the bonding between adhesive systems and dentine substrates remains unsatisfactory. The factors influencing the bonding quality include the composition of the adhesive system, its structural and morphological features, as well as the composition of the dentine substrate.^18^ In addition, collagen has fundamental importance in determining the longevity of adhesives,^11^ since the demineralisation of dentine is necessary for the formation of a hybrid layer exposing the collagen fibre network.^13,31^

Conventional adhesive systems use phosphoric acid to condition dentine, but these adhesives also promote a wide range of collagen fibre exposure, which may exceed the infiltration capacity of the adhesive systems.^7,14,17^ Self-etching adhesive systems have been introduced in dentistry,^13^ and recently, universal adhesive systems were introduced to minimise the required clinical steps. According to the manufacturer, these adhesives can adhere to the dental tissue by acid etching and self-etching techniques,^7^ through the acidic monomers present in them, and simultaneously infiltrate the collagen network.^8^

The collagen network that is not involved by the adhesive system is susceptible to hydrolytic degradation and the action of collagenolytic enzymes and MMPs (metalloproteases).^7,23^ The degradation process of collagen fibres causes restoration infiltration, secondary caries and sensitive teeth, causing the failure and poor longevity of the restorative procedure.^3,19^ To control the effects of the incomplete involvement of collagen fibrils by the adhesive system, MMPs inhibitors are commonly used as well as the chlorhexidine,^25,30^ once the inhibitor application on dentine surface after acid etching could result in improvement of the integrity and stability of the tooth restoration.^5,12^

However, data on the efficiency of these universal adhesive systems associated with MMP inhibitors in the bond strength of the restorative procedures are scarce in the literature. Therefore, the purpose of this study was to evaluate the influence of the preapplication of 2% chlorhexidine gluconate on the immediate bond strength of a universal adhesive system with dentine subjected to different bonding protocols 24 h after bonding. The null hypothesis tested was that the preapplication of chlorhexidine gluconate would not cause changes in the immediate bond strength of the universal adhesive system with dentine, regardless of the adhesive protocol adopted.

## Materials and Methods

### Specimen Treatment

The materials used in this study are listed in Table 1. The study was approved by the local Research and Ethics Committee. A total of 20 human molars from different individuals, extracted for orthodontic or periodontal reasons, were used in this study. All the teeth that exhibited fractures, cracks or clinical signs of caries were excluded.

**Table 1:** Trademark, classification and composition of materials used in this study

Material	Classification	Composition
Single Bond Universal (3M Oral Care)	Adhesive system	MDP, Bis-GMA, HEMA, photoinitiators, dimethacrylate, water, ethanol, silane
Filtek Z250 XT (3M Oral Care)	Resin composite	Inorganic fillers (60%), Bis-GMA, UDMA, Bis-EMA, zirconia/silica nanofillers

MDP, 10-Methacryloyloxydecyl dihydrogen phosphate; Bis-GMA, bisphenol-A diglycidyl ether dimethacrylate; HEMA, 2-hydroxyethyl methacrylate; UDMA, urethane dimethacrylate; Bis-EMA, ethoxylated bisphenol-A glycol dimethacrylate.

**Fig 1:**
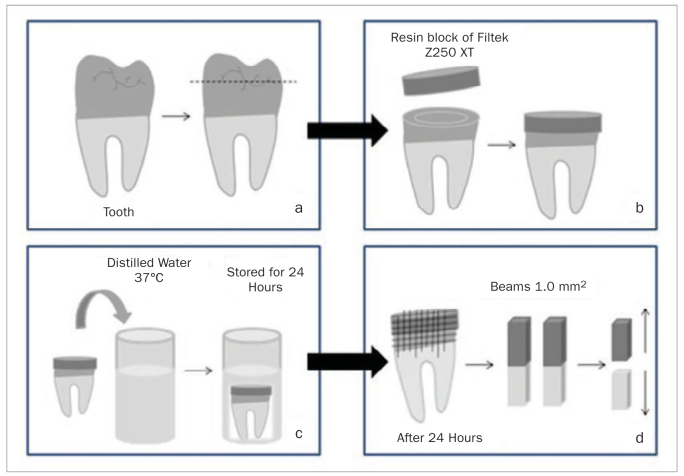
Experimental specimen preparation. (a) Occlusal surfaces of the teeth were ground flat to expose dentine. (b) Bonding process involving the Filtek Z250 XT resin composite. (c) The teeth were stored for 24 h in distilled water at 37 °C. (d) Specimens were sectioned perpendicularly to the adhesive interface to produce beams with an adhesive area of approximately 1.0 mm² for microtensile bond strength testing.

The occlusal surfaces of all the teeth were ground flat with #180, #320, and #600 grit silicon carbide abrasive papers (Extec, Enfield, CT, USA) under running water in an automatic polishing machine (APL-4; Arotec, São Paulo, SP, Brazil) to remove the enamel and expose the flat dentine surface (Fig 1a). The teeth were randomly distributed into four groups (n = 5)^10,27^ according to the surface treatments described next.

In the SBU group, the Single Bond Universal adhesive system (3M Oral Care, St. Paul, MN, USA) was actively applied on the dentine surfaces for 20 s without previous acid etching and followed by air jet for 5 s and photoactivation for 20 s with an Ultraled light-curing unit (Dabi Atlant, Ribeirão Preto, SP, Brazil). Increments of 2 mm thickness of the Filtek Z250 XT resin composite (3M Oral Care) were applied on the dentine surface and light-cured for 20 s until a 6 mm resin block was obtained (Fig 1b).

In the SBUPA group, the teeth were etched with 37% phosphoric acid (3M Oral Care) for 15 s, washed and dried with paper towels without dehydrating the dentine. Subsequently, the same restorative procedure as that reported for the above group was performed.

The dentine surface of the SBUCG group received the same treatment as that of the SBU group, but the dentine was preconditioned with 0.5 ml of 2% chlorhexidine gluconate (Aphoticario, Araçatuba, SP, Brazil) actively applied in the dentine using a microbrush for 60 s. The excess solution was removed using paper towels.

In the SBUPACG group, the dentine surface was conditioned with 2% chlorhexidine gluconate after being acid-etched with 37% phosphoric acid and before applying the adhesive system.

After the bonding process, all the teeth were stored in distilled water at 37°C for 24 h (Fig 1c). After this period, the teeth were sectioned perpendicular to the adhesive-tooth interface with a low-speed diamond saw (Isomet 1000, Buehler, Lake Bluff, IL, USA) under water cooling to obtain beams with an adhesive area of approximately 1.0 mm² (Fig 1d).^29^ It was stipulated that six beams from the middle region for each specimen would be obtained, totalling 30 beams for each experimental group.

### Microtensile Bond Strength Analysis

The ends of the stick-like specimens were fixed with a cyanoacrylate adhesive (Super Bonder gel; Henkel Corp, Rocky Hill, CT, USA) to a metallic testing apparatus and individually subjected to microtensile testing using an OM100 machine (Luzerna, SC, Brazil). The machine was operated at a crosshead speed of 0.5 mm/min to evaluate the microtensile bond strength values (MPa) according to the following formula^20^:

RU = F/A, where Ru is the bond strength, F the maximum force, and A the area of the adhesive interface (mm²). The beams that suffered premature loss were assigned a value of zero for the bond strength.

Representative samples of the experimental groups were coated with gold (BAL-TEC SCD 050; Balzers, Balzers, Liechtenstein) and analysed using a scanning electron microscope (SEM-JSM5600LV; JEOL, Tokyo, Japan) to characterise the surfaces subjected to the different bonding protocols.^15^ The bond strength data were subjected to ANOVA and Sheffé´s least statistically significant difference tests (α = 0.05).

## Results

The results of the ANOVA test for the bond strength values indicated no statistically significant differences among all the analysed groups (p = 0.3678, Table 2). Despite the non-statistically significant difference, the SBUPA group showed higher bond strength values compared to those of the SBUCG group, which showed the highest number of beams premature loss (Table 3).

**Table 2:** Mean (standard deviation, MPa) values of microtensile bond strengths of adhesive system on dentine subjected to different bonding protocols

Bond strength	SBU group	SBUPA group	SBUCG group	SBUPACG group
	29.26 ± 9.06 A	32.07 ± 11.70 A	20.83 ± 8.72 A	29.41 ± 11.31 A

SBU group: Single Bond Universal without acid etching; SBU_PA_ group: 37% phosphoric acid + Single Bond Universal; SBU_CG_ group: 2% chlorhexidine gluconate + Single Bond Universal; and SBU_PACG_ group: 37% phosphoric acid + 2% chlorhexidine gluconate + Single Bond Universal. Different uppercase letters indicate statistically significant differences (p < 0.05).

**Table 3:** Incidence (numbers) of the beams exhibiting premature loss

	SBU group	SBU_PA_ group	SBU_CG_ group	SBU_PACG_ group
Premature Loss	1	0	4	2

SBU group: Single Bond Universal without acid etching; SBU_PA_ group: 37% phosphoric acid + Single Bond Universal; SBU_CG_ group: 2% chlorhexidine gluconate + Single Bond Universal; and SBU_PACG_ group: 37% phosphoric acid + 2% chlorhexidine gluconate + Single Bond Universal.

## Discussion

Universal adhesive systems can be used with or without previous acid etching, and with wet or dry dentine, depending on the restorative technique and dentine substrate.^21^ Chlorhexidine has been widely used in dentistry because of its antimicrobial properties, substantivity and effect on the adhesive interface longevity.^1,2^ Chlorhexidine has been shown to maintain the quality of the dentine substrate by inhibiting the collagenolytic activity of the MMPs in the hybrid layer.^4^ The results presented here show that the chlorhexidine solution associated with a universal adhesive system did not influence the bond strength values (Table 2), and the null hypothesis of the study was accepted.

The Single Bond Universal adhesive system is composed of a 10-MDP functional monomer, exhibiting a higher chemical bonding potential to crystals of hydroxyapatite, promoting the formation of highly insoluble calcium salts and a satisfactory and stable adhesion.^16^ The addition of this functional monomer to the adhesive system was carried out to prevent differences in the demineralisation depth caused by the acid etching pretreatment and penetration of the conventional adhesive system, and prevent the hydrolytic degradation of the collagen fibres.^22,24^ The action of this adhesive system results in superficial demineralisation and penetration of the resinous monomer concomitant into the substrate, which can explain the satisfactory bond strength observed in this study for the adhesive system with the dentine substrate (Table 2). It is important to relate that careful was taken about the moisture of the dentine, which was controlled during all procedures for not causing shrinkage of the collagen fibrils.

**Fig 2:**
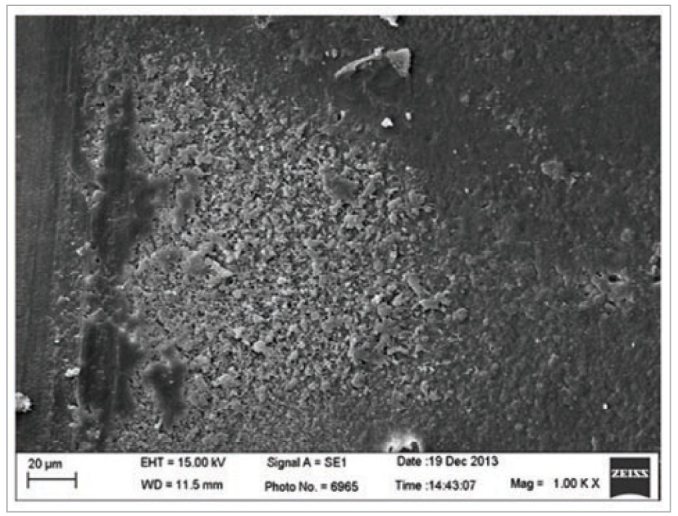
Representative image of the dentine without acid etching.

**Fig 3:**
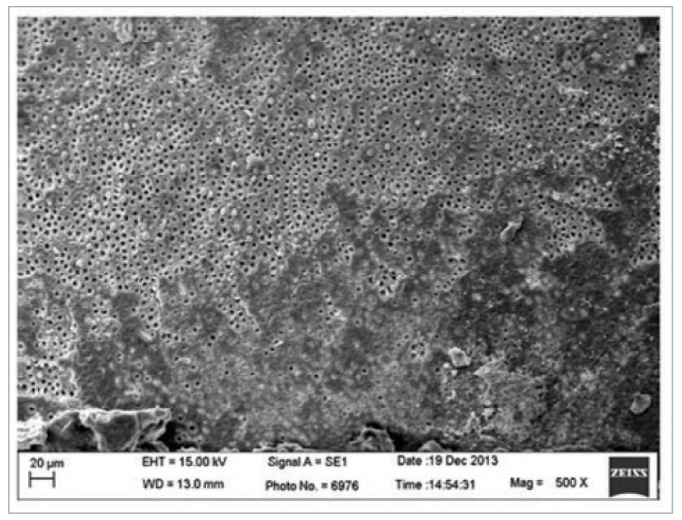
Representative image of the dentine conditioned with 37% phosphoric acid.

As previously described, collagen fibrils are fundamentally important in determining the longevity of adhesive procedures.^11^ Acid etching is more sensitive to the moisture of the exposed network collagen fibrils before the application of the adhesive system than the self-etching technique.^9^ The collagen fibrils collapse under the drying of the dentine substrate and the unsatisfactory penetration of the adhesive, and the involvement of the collagen fibril mesh results in lower bond strengths.^9^ However, the results of this study showed no statistically significant difference in the bond strengths between phosphoric acid etching and the acidic monomers from the self-etch universal adhesive system in dentine substrate conditioning (Table 2). This could be due to the high diffusion rate of the adhesive system in the dentine tubules and collagen fibril mesh. In addition, the aggressiveness of the erosive nature of phosphoric acid could contribute to collagen fibril exposure, resulting in the infiltration of the collagen network by the monomers (Figs 2 and 3).^9^

**Fig 4:**
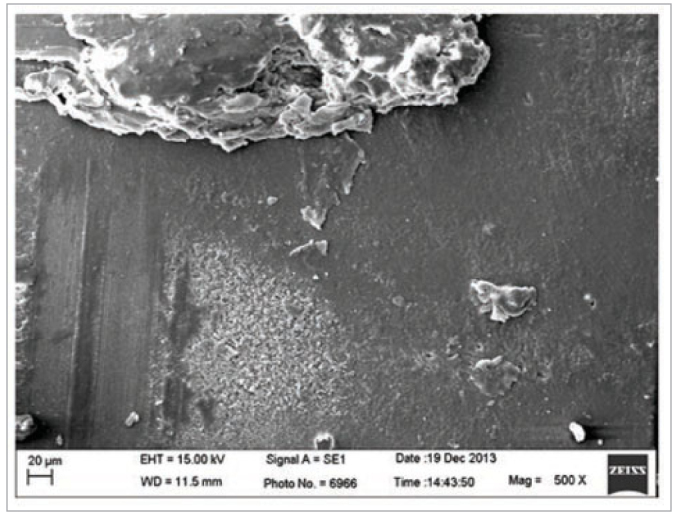
Representative image of the dentine conditioned with 2% chlorhexidine gluconate.

**Fig 5:**
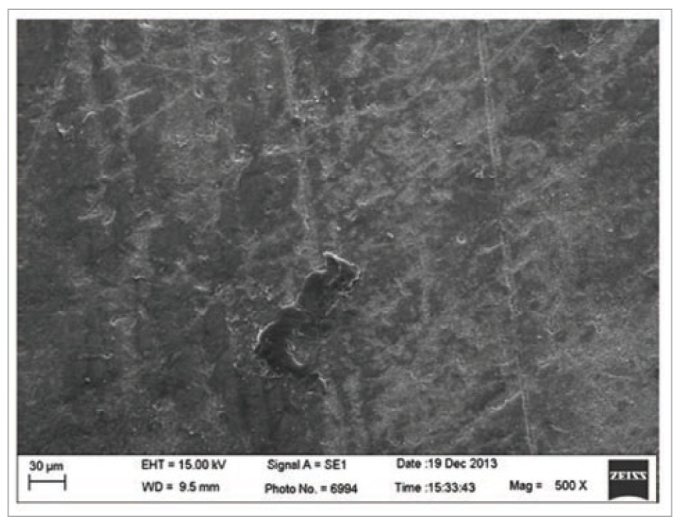
Representative image of the dentine conditioned with 37% phosphoric acid and 2% chlorhexidine gluconate.

Despite the non-statistical differences between the experimental groups, the group where the dentine was subjected to conditioning with the 2% chlorhexidine gluconate solution showed a lower numeral bond strength values than that for the dentine conditioned with phosphoric acid (Table 2). Chlorhexidine has a strong cationic charge, which can strongly bind to anionic molecules, such as the phosphate present in hydroxyapatite, and influence the phosphate/calcium ratio.^26,32^ The reaction between phosphate and chlorhexidine solution results in precipitation,^6^ which could act as a physical barrier reducing the maximum contact between the adhesive material and tooth surface (Figs 4 and 5). This may be the mechanism underlying the higher premature loss in dentine groups subjected to conditioning with chlorhexidine (Table 3).

Moreover, because the chlorhexidine was not washed off the dentine, it is hypothesised that the dentine tubules are physically occupied and occluded by the chlorhexidine molecules and debris remaining on the dentine.^28^ This could interfere in the satisfactory infiltration of the resinous monomers of the adhesive, decreasing the area of contact between the adhesive system and dental substrate.^26^

The inability to simulate biological changes such as chemical attack by acids and enzymes, low specimens per group, analysis of bond strength with no aging procedure, as well as the use of only one universal adhesive system and solution concentration can be considered the major limiting factors of this study. The 2% chlorhexidine gluconate solution did not influence the adhesive immediate bond strength, but further laboratory and clinical studies should be performed to clarify the influence of chlorhexidine on dentine. These studies may include permeability analysis, investigation of the mechanical properties of the dentine and restorative substrate, as well as hardness and elastic modulus tests with associated longitudinal analyses.

## Conclusions

Based on results of the present study, it can be concluded that the preapplication of 2% chlorhexidine gluconate did not reduce the immediate bond strength of the Single Bond Universal adhesive system under different bonding protocols.
